# A survey to assist in targeting the adults who undertake risky behaviours, know their health behaviours are not optimal and who acknowledge being worried about their health

**DOI:** 10.1186/1471-2458-13-120

**Published:** 2013-02-08

**Authors:** Anne W Taylor, Kay Price, Simon Fullerton

**Affiliations:** 1 Population Research and Outcome Studies, University of Adelaide, Level 3, 122 Frome Street, Adelaide, South Australia, 5000, Australia; 2 Associate Professor Kay Price, School of Nursing and Midwifery, University of South Australia-City East Campus, GPO Box 2471, Adelaide, SA, 5001, Australia

**Keywords:** Worry, Risk factors, Perception, Australia, Health promotion

## Abstract

**Background:**

Research indicates that those who are worried about their health are more likely to change their in-appropriate behavioural-related risk factors. A national survey was undertaken to determine adults who correctly perceive and actually undertake in-appropriate behavioural-related risk factors (smoking, physical activity, alcohol intake, fruit and vegetable consumption, weight and psychological distress) and are worried about their health.

**Methods:**

Australian 2010 CATI survey of 3003 randomly selected adults. Perception and self-reported levels of each risk factor, and whether they worried that the level was affecting their health were assessed using univariate and multivariate analyses.

**Results:**

The comparisons between perception of healthy behaviour and actual behaviour varied for each risk factor with 44.1% of people in the un-healthy weight range and 72.9% of those eating less than sufficient fruit and vegetables having the perception that their behaviour was healthy. The demographic and other related variables in the multivariate analyse for each risk factor varied considerably. For example the variables in the final multivariate model for smokers who were worried about their risk factor were markedly different to the other risk factor models and 45 to 54 year olds were more likely to be included in the final models for nearly all of the risk factor analyses.

**Conclusion:**

By limiting this analyses to those who are acknowledging (correctly or otherwise) that their perception of behaviour is making their health worse, this study has shown that the profile for each risk factor varies considerably. As such, evidence suggests specific targeted programs are required rather than a broad brush approach.

## Background

Understanding what makes some people undertake risky behaviours, and others not to, is somewhat perplexing and the basis of much research. Various social cognition theories and models associated with behaviour change (such as stages of change, risk perception trans-theoretical model, theory of planned behaviour and health belief model) have been formulated to assist in understanding this phenomenon of undertaking risky behaviours [1]. As theorised by many models (see for example [2-4]), acknowledgement that a risk to one’s health exists is a procurer to behaviour change. This understanding is based on the principle that if one does not believe they are at risk then they are unlikely to perceive a need to change behaviour. This perceived risk can have a positive relationship on seeking health information [2].

Studies have shown that worry is also related to personal action [5] and has an important role in helping people make decisions. Although worry has negative connotations it is an important step in endorsing protection against harm [5] and motivating action towards appropriate health promotion behaviours [6]. Worry has also been shown to be related to the need for an increased amount of information [7] and more positive attitudes towards, and intentions to make, behavioural change [8].

Research has been undertaken on the relationship between perceived risk and worry associated with health effects and the resultant change in risky behaviours [2,8,9]. Cameron & Reece [2] argue that perceived risk and worry ‘reflect two parallel systems of information processing’. As argued by many there are two components – a reasoned component (risk) and an emotional component (worry) [9] and that worry can have the strongest predictive nature more than perceived risk [2,10]. Others have argued that risk perception is but a judgement about worry [9]. Either way, admitting a perceived risk and having a degree of worry about the situation are highlighting a desire for change, a willingness to listen to information provided and a readiness to take action [7,11,12].

In most theories and models, especially those highlighting cognitive processes, the more one is ‘under threat’ the more one is likely to accept advice/recommendations [11,13]. Previous research has shown that higher levels of worry predicts a more positive attitude and intention to change but this was more likely to occur for those with the worst-levels of behaviours [8].

In a unique Australian study [14], questions on four key health-related behavioural risk factors (physical activity, smoking status, alcohol consumption, fruit and vegetable consumption as an indication of good nutrition), and two health status outcomes closely related to behaviour and behaviour modification (body mass index (BMI) as an indication of adiposity, and Kessler 10 (K10) as a measure of psychological distress), were assessed together with perception of whether the respondents believed each of their risk factors was at a desired level. Each respondent who perceived they were not at an optimal level were then asked if they worried that the shortfall was affecting their health. This has allowed analysis to be undertaken to assist in determining appropriate interventions based on people’s perception of risk, their actual behaviour/risk factor and knowledge of how correct the perception was when compared with actual behaviour.

In the endeavour to change inappropriate or risky behaviours of the population, mass media campaigns and interventions based on increased communication and information exchange are often the preferred intervention strategy [15,16], but improved targeting information is required. If targeted properly, addressing the consumer’s needs, interests and motives, the chance of a successful behaviour change is enhanced [17]. While socio-demographic data provide meaningful evidence of who should be targeted, demographic characteristics are limited in their ultimate changeability [1]. Additional evidence, such as the variables provided in this analysis, are more readily amenable to change hence provide important evidence for health promotion experts.

In this paper we highlight the different profile of risk behaviours and their relationship to perception of risk and actual behaviour. We hypothesise that people who are worried about their health are more likely to change their unhealthy behaviours [13] and as such we provide, for each of the six key risk factors, a multivariate analysis of demographic, socio-economic and other health-related variables.

## Method

A ‘Novel approach to Influencing Self Care’ project [14] was funded through the Australian Federal Government Sharing Health Care Initiative. The aim of the mixed methods study was to inform health professionals and policy makers of the best strategies to support targeted groups of people with chronic conditions to more effectively manage their health. Data used in the analysis of this paper were obtained from a national survey - Stage 3 of the ‘Novel approach to Influencing Self Care’ study. The questionnaire was developed from previous stages of the study that included detailed profiles of respondents of the North West Adelaide Health Cohort Study (Stage 1) [18] and semi-structured interviews (Stage 2) [14]. The national survey (Stage 3) – the focus of this stand-alone analysis - was designed to gather information about what was driving decision-making on an everyday basis for people living with and without chronic conditions, as well as what risky behaviours they engage in and if they are aware of this risk. While the survey was used to explore a range of other health-related issues, only selected variables from the national survey were used in these analyses.

### Data collection

All households in Australia with a connected telephone and the telephone number listed in the Australian Electronic White Pages (EWP) were eligible for selection. Within each household contacted, a random person (the person, aged 18 years or over, who was last to have a birthday) was selected. There was no replacement for non-contactable persons. On average, interviews took 15 minutes to complete. In an endeavour to increase response rates, a letter outlining the purpose of the study was sent to selected households. Data collection was undertaken by a contracted agency using trained interviewers in April and May, 2010. Interviews were conducted using Computer Assisted Telephone Interview (CATI) methodology.

A minimum of 10 call-backs were made to telephone numbers selected for interview. Different times of the day or evening were scheduled for each call-back. If the person could not be interviewed immediately they were re-scheduled for interview at a time suitable to them. Replacement interviews for persons who could not be contacted or interviewed were not permitted. Ten percent of each interviewer’s work was randomly selected for validation by the supervisor.

An initial sample of 10,000 telephone numbers was drawn. Sample loss of 3,138 occurred due to non-connected numbers (n = 2,641), non-residential number (n = 276), fax/modem connections (n = 206) and ineligible households (n = 15). The overall sample response rate was 43.8%.

### Questionnaire

Respondents were initially asked their perception of their risk factor (do you think you exercise enough; do you eat a balance diet; do you drink more alcohol than is good for you or than you should; do you think you are overweight, underweight, OK weight; and do you think you worry or stress more than is good for you). If they answered negatively for exercise or diet, positively for alcohol or stress, or responded underweight or overweight regarding weight, they were then asked whether they worried about it (eg does it worry you that not exercising enough may affect your health). Current smokers and ex-smokers were also asked if they worry that their previous/current smoking could affect their health.

Risk factor questions were asked towards the end of the questionnaire and were: how many times a week physical activity of at least 30 minutes was undertaken (insufficient activity defined as less than 150 minutes of physical activity per week), how many serves of vegetables and fruit per day consumed (with respondents deemed to not be eating the required number if they reported less than five serves of vegetables or two serves of fruits per day) [19]; how often and on how many days alcohol was consumed (with risky levels for men, defined as consuming seven or more drinks on any one occasion or alcohol consumption four or more times per week; for women, defined as consuming five or more drinks on any one occasion or alcohol consumption four or more times per week) [20]; BMI (self-reported height and weight) [21]; psychological distress using the Kessler 10 (by receiving a score of 22 or higher on the Kessler 10 instrument) [22]; and smoking status. These questions have all been tested for validity and reliability in the Australian CATI setting [23].

The value of this study is the wide range of ancillary health-related questions asked in the survey. These other questions included in the analysis were based on issues brought up in the focus group discussions and these covered three key concepts - ‘perception of health’ and ‘health service use’ and ‘health action’. Perceptions of health questions were overall health status, whether life was affected by health conditions, how often pain stopped activity, how often the respondent had enough energy, how often they felt angry about their health, and whether they cared about their health. Health service use questions were use of complementary and alternative medicines, doctor visits in the previous year, and other health professional visits in the previous year. Health action related questions were how often they had to adjust pace because of health, whether they did things to reduce their stress, whether they tried to stay connected with people, and if they had ever used trial and error. ‘Trial and error’ is a decision-making strategy that is personal and purposefully implemented to assist an individual to make sense of what is/is not possible for them to do in everyday circumstances. Decisions people make are not necessarily made being mindful of how their decisions will impact on their future health status [24].

Demographic questions asked included age, sex, marital status, work status, country of birth, highest education level obtained, housing status, and annual household income.

### Statistical methods

Raw data from the CATI system were analysed using SPSS Version 18.0 and Microsoft Excel. The data were weighted by age and sex to reflect the structure of the Australian population 18 years and over using the Australian Bureau of Statistics 2006 Estimated Residential Population. The weights reflect unequal sample inclusion probabilities and compensate for differential non-response.

Initially, a prevalence estimate for each risk factor was produced for the whole sample. In addition, a comparions between actual risk factor status and perceived risk (healthy, unhealthy) was undertaken using data from all participants. As the focus of this paper was on perceived unhealthy behaviours, no further analyses were undertaken for those whose perception was that they were undertaking the behaviour at a healthy level. Actual risk factor status was then assessed against worry status (worried, not worried). To determine the population most likely to change their behaviour (based on the fact that they believe they are at risk, they actually are in the risky category plus they are worried about the affect of the risk factor on their health – our target population), univariable analyses using chi-square tests were employed to compare differences for each of the six behaviours/risk factors. Six separate multivariable logistic regression models were subsequently developed. As recommended by Hosmer and Lemeshaw [25], all variables with a p-value <0.25 at the univariable level, were included in the initial multivarialbe model in order to ascertain independently associated factors. Final models were obtained using backward stepwise elimination of non-significant variables based on the log likelihood ratio tests. A p-value less than 0.05 was regarded as statistically significant.

The research was carried out following approval from the University of South Australia Human Research Ethics Committee, which are guided by the Australian code for the responsible conduct of research & the National Statement on Ethical Conduct in Human Research 2007.

## Results

Overall 3001 interviews were conducted with 48.7% being with males. The mean age was 44.9 years (SD 15.1). Table 1 contains the demographic characteristics of the complete sample.

**Table 1:** Socio-economic and demographic characteristics of respondents, Australia, 2010

**Demographic and socioeconomic profile of respondents**			
	**n**	**%**	**(95%CI)**
**Sex**			
Male	1463	48.7	(46.9–50.5)
Female	1540	51.3	(49.5–53.1)
**Age group**			
65+ years	528	17.6	(16.3–19.0)
55 to 64 years	432	14.4	(13.2–15.7)
45 to 54 years	558	18.6	(17.2–20.0)
35 to 44 years	589	19.6	(18.2–21.1)
18 to 34 years	897	29.9	(28.3–31.5)
**Number of adults in household aged 18+ years**			
One	332	11.1	(10.0–12.2)
Two	1688	56.2	(54.4–58.0)
Three or more	983	32.7	(31.1–34.4)
Country of birth*			
Australia	2349	78.2	(76.7–79.6)
UK / Ireland	184	6.1	(5.3–7.0)
Other	469	15.6	(14.4–16.9)
**Aboriginal or Torres Strait Islander**			
No	2287	97.4	(74.6–77.7)
Aboriginal / Torres Strait Islander	58	2.5	(1.5–2.5)
**Family structure**			
Family and children	1574	52.4	(50.6–54.2)
Adult living alone	284	9.5	(8.5–10.6)
Adult living with partner - no children	769	25.6	(24.1–27.2)
Adults living together - related / unrelated	343	11.4	(10.3–12.6)
Other	30	1.0	(0.7–1.4)
**Marital status**			
Never Married	651	21.8	(20.3–23.3)
Married/living with partner	1999	67.0	(65.2–68.6)
Separated/divorced	189	6.3	(5.5–7.2)
Widowed	147	4.9	(4.2–5.8)
**Employment status**			
Full time employed	1287	42.9	(41.1–44.6)
Part time employed	564	18.8	(17.4–20.2)
Unemployed	105	3.5	(2.9–4.2)
Economically inactive (Home duties, student, retired, unable to work, other)	1046	34.8	(33.2–36.6)
**Highest education level obtained**			
No schooling to secondary	1383	46.1	(44.3–47.9)
Trade, certificate, diploma	758	25.2	(23.7–26.8)
Degree or higher	813	27.1	(25.5–28.7)
**Undertake volunteer activities**			
Yes	1018	33.9	(32.2–35.6)
No	1985	66.1	(64.4–67.8)
**Provide long term care**			
Yes	771	25.7	(24.2–27.3)
No	2232	74.3	(72.7–75.8)
**Gross annual household income**			
$80,000 or more	1136	37.8	(36.1–39.6)
$40,001-$80,000	698	23.2	(21.8–24.8)
Up to $40,000	575	19.1	(17.8–20.6)
Not stated	594	19.8	(18.4–21.3)
**Household money situation**			
Spending more money than receive	125	4.2	(3.5–4.9)
Just enough money to get through to next pay day	549	18.3	(16.9–19.7)
Some money left over each week but spend it	184	6.1	(5.3–7.0)
Save a bit every now and then	1441	48.0	(46.2–49.8)
Save a lot	590	19.6	(18.3–21.1)
Don’t know	87	2.9	(2.4–3.6)
Refused	27	0.9	(0.6–1.3)
Dwelling type*			
Owned or being purchased	2458	82.1	(80.7–83.4)
Rented from government housing	86	2.9	(2.3–3.5)
Rented privately	398	13.3	(12.1–14.6)
Community housing / Retirement village/other	52	1.7	(1.3–2.3)
**Total**	**3003**	**100.0**	

Note: The weighting of the data can result in rounding discrepancies or totals not adding. *Don’t know category not included.

Table 2 details, for the whole sample, the overall prevalence estimates associated with each risk factor. In total, 18.2% of respondents had at least four of these six risk factors.

**Table 2:** Prevalence estimates associated with each risk factor, Australia, 2010

	**n**	**%**	**(95% CI)**
**BMI**			
Underweight	79	3.0	(2.4–3.8)
Normal	1059	40.5	(38.6–42.4)
Overweight	924	35.3	(33.5–37.2)
Obese	553	21.1	(19.6–22.7)
**Fruit and vegetable consumption**			
At least 2 & 5 serves per day	266	8.9	(7.9–9.9)
Less than 2 & 5 serves per day	2737	91.1	(90.1–92.1)
**Physical activity**			
Sufficient activity	1592	53.1	(51.3–54.8)
No activity/activity but not sufficient/don’t know	1409	46.9	(45.2–48.7)
**Smoking**			
Non-smoker	1390	46.3	(44.5–48.1)
Ex-smoker	1084	36.1	(34.4–37.8)
Current smoker	529	17.6	(16.3–19.0)
**Short term alcohol risk**			
Non-drinker	685	22.8	(21.3–24.4)
Low risk	1463	48.8	(47.0–50.5)
Risky	725	24.2	(22.7–25.7)
High risk	128	4.3	(3.6–5.1)
**Psychological distress**			
Low	1872	62.6	(60.8–64.3)
Moderate	762	25.5	(23.9–27.1)
High	256	8.6	(7.6–9.6)
Very high	101	3.4	(2.8–4.1)
**Number of risk factors**			
None	53	2.0	(1.6–2.7)
One	382	14.7	(13.3–16.1)
Two	810	31.1	(29.3–32.9)
Three	884	33.9	(32.1–35.8)
Four	386	14.8	(13.5–16.3)
Five	76	2.9	(2.3–3.6)
Six	14	0.5	(0.3–0.9)
Total	3003	100.0	

Table 3 highlights, for the whole sample, the actual risk factor category by the perception of risk (healthy or unhealthy) associated with individual behaviour. The proportion in the correct/normal risk category and whose perception matched, ranged from 91.1% for fruit and vegetable consumption to 46.4% for psychological distress. Conversely, those who were actually in the risk category but whose perception was incorrect (believing the risk factor was in the healthy range) varied from 11% for psychological distress to 72.9% for fruit and vegetable consumption meaning that over 70% of respondents believed they were eating a balanced healthy diet when their actual consumption of fruit and vegetables (as an indicator of a healthy diet) was less than the recommended two and five serves of fruit and vegetables per day. Proportions ranged from 27.1% for inadequate serves of fruit and vegetable consumption to 89.0% for psychological distress for those in the risky category, whose perception and actual behaviour matched. Smoking is not included in this table as questions related to smoking were limited to current smoking and worry status, rather than perception of risk, with all current and ex-smokers deemed to be at risk.

**Table 3:** Risk factor perception versus actual behaviour, Australia, 2010

	**Perception - healthy**			**Perception - unhealthy**		
**Actual measure**	**n**	**%**	**(95**% **CI)**	**n**	**%**	**(95%CI)**
**BMI**						
Normal	958	90.5	(88.6–92.1) ↑	101	9.5	(7.9–11.4) ↓
Underweight /Overweight / Obese	688	44.1	(41.7–46.6) ↓	870	55.9	(53.4–58.3) ↑
**Fruit and vegetable consumption**						
Recommended amount	243	91.1	(87.1–94.0) ↑	24	8.9	(6.0–12.9) ↓
Less than recommended amount	1995	72.9	(71.2–74.5) ↓	742	27.1	(25.5–28.8) ↑
**Physical activity**						
Sufficient activity	1003	63.0	(60.6–65.3) ↑	589	37.0	(34.7–39.4) ↓
No activity/activity but not sufficient/don’t know	364	25.9	(23.6–28.2) ↓	1044	74.1	(71.8–76.4) ↑
**Short term alcohol risk**						
Non-drinker/no risk	1694	78.9	(77.1–80.5) ↑	454	21.1	(19.5–22.9) ↓
Risky/high risk	285	33.4	(30.3–36.6) ↓	568	66.6	(63.4–69.7) ↑
**Psychological distress**						
Low/ Moderate	1223	46.4	(44.5–48.4) ↑	1411	53.6	(51.6–55.5) ↓
High /Very high	39	11.0	(8.1–14.6) ↓	318	89.0	(85.4–91.9) ↑

↑↓ Statistically significantly higher or lower than other group (p < 0.05).

Table 4 highlights the worry status and risk factor status only for those respondents who believe their risk profile is not optimal (that is, regardless of their actual behaviour, their perception was that they were undertaking the actual behaviour at an unhealthy level (those in the right hand column of Table 3)). When comparing those who have the risk factor significant differences were apparent for all risk factors, except for short-term alcohol risk. The proportion who are actually in the normal range but who still worry about the risk factor affecting their health ranged from 43.4% for normal BMI to 98.6% of those eating two and five serves of fruits and vegetables per day. The proportion who were at risk and correctly worried about it (our target population) ranged from 48.0% for alcohol to 92.2% for psychological distress.

**Table 4:** Risk factors status (for those whose perception is of unhealthy behaviour), by worry status, Australia, 2010

	**Worried**			**Not worried**		
**Actual measure**	**n**	**%**	**(95**% **CI)**	**n**	**%**	**(95**% **CI)**
**BMI**						
Normal	44	43.4	(34.2–53.2) ↓	57	56.6	(46.8–65.8) ↑
Underweight /Overweight / Obese	612	70.3	(67.2–73.3) ↑	258	29.7	(26.7–32.8) ↓
**Fruit and vegetable consumption**						
Correct amount	23	98.6	(83.7–99.9)	1	1.4	-
Less than correct amount	564	76.1	(72.9–79.0) ↓	178	23.9	(21.0–27.1) ↑
**Physical activity**						
Sufficient activity	439	74.5	(70.9–77.9) ↓	150	25.5	(22.1–29.1) ↑
No activity/activity but not sufficient/don’t know	849	81.3	(78.8–83.5) ↑	196	18.7	(16.5–21.2) ↓
**Smoking**						
Non/ex-smoker	525	67.5	(64.2–70.7) ↓	252	32.5	(29.3–35.8) ↑
Current smoker	421	79.6	(75.9–82.8) ↑	108	20.4	(17.2–24.1) ↓
**Short term alcohol risk**						
Non-drinker/no risk	230	50.6	(46.0–55.2)	224	49.4	(44.8–54.0)
Risky/high risk	272	48.0	(43.9–52.1)	296	52.0	(47.9–56.1)
**Psychological distress**						
Low/ Moderate	960	68.0	(65.5–70.4) ↓	451	32.0	(29.6–34.5) ↑
High /Very high	295	92.9	(89.5–95.2) ↑	23	7.1	(4.8–10.5) ↓

↑↓ Statistically significantly higher or lower than other group (p < 0.05).

Table 5 highlights the results of the multivariate analysis for all six risk factors with each column showing the odds associated with the risk factor for respondents who were worried about the individual risk factor. There was a range of conflicting results for different risk factors. For example, being married or living with a partner meant that you were less likely to have high psychological distress (OR 0.65) but more likely to have an increased BMI (OR 1.64). This pattern was also found for example for work status (with increased risk for physical inactivity and alcohol for the unemployed and lower risk for BMI for the unemployed), dwelling type (increased risk for smoking and psychological distress and lower risk for physical inactivity for those renting from the government), household income (higher risk for BMI and psychological distress and lower risk for physical activity for the middle income group), visits to a doctor in the last year (increased risk for BMI and lower risk for smoking), visits to other health professional in the last year (with increased risk for fruit and vegetable consumption and alcohol and lower risk for smoking) and use of trial and error (with increased risk for BMI and fruit and vegetable consumption and lower risk for alcohol). Education level was also different across risk factors with increased odds for those with a degree or higher and who are not eating enough fruit and vegetables (OR 2.06) and for insufficient physical activity (OR 1.53) whilst those with trade, certificate or diploma level of education had a lower risk for smoking (OR 0.68) and short term alcohol risk (OR 0.61).

**Table 5:** Multivariate analysis of factors associated with participants who worry about their behaviour and who thought their behaviour unhealthy, by risk factor, Australia, 2010

	***BMI***	***F & V***	***PA***	***Smoking***	***Short term alcohol risk***	***High Psychological distress***
**Number of people in each behaviour/worry group**	612/971	564/765	849/1633	421/1306	272/1022	295/1728
**% in each behaviour/worry group (95**% **CI)**	63.0 (60.0–66.0)	73.7 (70.5–76.7)	51.9 (49.5–54.4)	32.2 (29.7–34.8)	26.7 (24.0–29.5)	17.1 (15.4–18.9)
***Sex***						
*Male*					1.00	
*Female*					**1.41 (0.04)**	
***Age***						
*65+ years*	1.00	1.00	1.00	1.00	1.00	1.00
*55 to 64 years*	**1.82 (0.02)**	2.04 (0.13)	1.28 (0.29)	**2.80 (<0.01)**	1.35 (0.39)	1.61 (0.17)
*45 to 54 years*	**2.21 (<0.01)**	**3.30 (0.01)**	1.30 (0.25)	**3.94 (<0.01)**	**2.44 (0.01)**	**2.15 (0.03)**
*35 to 44 years*	1.48 (0.12)	2.23 (0.07)	**1.74 (0.02)**	**6.18 (<0.01)**	1.71 (0.13)	**2.17 (0.03)**
*18 to 34 years*	1.47 (0.13)	1.88 (0.15)	1.35 (0.19)	**8.60 (<0.01)**	1.80 (0.06)	**3.91 (<0.01)**
***Marital status***						
*Never Married*	1.00	1.00	1.00			1.00
*Married/living with a partner*	**1.64 (0.01)↑**	0.89 (0.63)	**0.66 (0.01)**			**0.65 (0.04)**
*Separated/divorced*	1.02 (0.95)	**0.43 (0.02)**	0.80 (0.41)			0.98 (0.96)
*Widowed*	1.37 (0.40)	1.10 (0.88)	0.89 (0.7)			0.61 (0.29)
***Education***						
*No schooling to secondary*		1.00	1.00	1.00	1.00	
*Trade, certificate, diploma*		1.09 (0.69)	1.23 (0.12)	**0.68 (0.02)**	**0.61 (0.01)**	
*Degree or higher*		**2.06 (0.01)**	**1.53 (<0.01)**	0.73 (0.10)	0.93 (0.70)	
***Work status***						
*Full time employed*	1.00		1.00	1.00	1.00	1.00
*Part time employed*	0.87 (0.38)		1.15 (0.33)	0.84 (0.32)	0.78 (0.30)	1.18 (0.46)
*Unemployed*	**0.32 (0.01)**		**2.86 (<0.01)**	0.83 (0.62)	**3.33 (0.01)**	1.12 (0.74)
*Economically inactive*	1.06 (0.71)		1.24 (0.15)	**0.60 (0.01)**	**1.48 (0.05)**	**1.80 (<0.01)**
***Dwelling***						
*Owned or being purchased*			1.00	1.00		1.00
*Rented from Government Housing*			**0.36 (0.01)**	**2.32 (0.01)**		**3.08 (<0.01)**
*Rented Privately*			1.19 (0.28)	**2.29 (<0.01)**		1.02 (0.93)
*Community/Retirement Village/ Other*			1.54 (0.31)	0.78 (0.72)		**5.80 (<0.01)**
***Country of Birth***						
*Australia*		1.00				
*UK/Ireland*		0.55 (0.19)				
*Other*		**0.59 (0.03)**				
***Household annual income***						
*>$80,000*	1.00		1.00			1.00
*$40,001-$80,000*	**1.81 (<0.01)**		**0.54 (<0.01)**			**1.60 (0.02)**
*<$40,000*	**1.66 (0.01)**		**0.65 (0.02)**			1.07 (0.79)
*Not stated*	**1.85 (<0.01)**		**0.55 (<0.01)**			0.83 (0.45)
***Overall quality of life***						
*Excellent/very good*			1.00	1.00		1.00
*Good*			**1.23 (0.09)**	1.15 (0.37)		**1.44 (0.05)**
*Fair/poor*			**1.55 (0.01)**	**1.97 (<0.01)**		**2.24 (<0.01)**
***Had complementary and alternative medicine***						
*No/don’t know*		1.00	1.00			1.00
*Yes*		**1.85 (<0.01)**	**1.56 (<0.01)**			**1.49 (0.02)**
***Life affected by health***						
*Activities limited/bedridden most of the time*	1.00					
*No problems /Can work & live normally day to day*	**1.55 (0.02)**					
***How often pain stops you doing what you want***						
*Always*		1.00				1.00
*Sometimes*		1.90 (0.11)				**0.53 (0.04)**
*Not/hardly at all*		**2.84 (0.01)**				**0.46 (0.02)**
***Doctor visits in past year***						
*None*	1.00			1.00		
*One to four times*	**1.65 (0.04)**			0.78 (0.31)		
*Five to ten times*	1.61 (0.07)			0.96 (0.87)		
*More than 10 times*	**1.99 (0.02)**			**0.34 (<0.01)**		
***Other health professional visits in past year***						
*None*		1.00		1.00	1.00	
*One to four times*		**1.94 (<0.01)**		**0.63 (<0.01)**	**1.72 (<0.01)**	
*Five to ten times*		0.88 (0.68)		**0.39 (<0.01)**	0.92 (0.75)	
*More than 10 times*		1.28 (0.52)		**0.41 (<0.01)**	0.98 (0.93)	
***How often have enough energy***						
*All/most of the time*				1.00		1.00
*Some of the time*				**1.47 (0.04)**		**2.10 (<0.01)**
*A little/none of the time*				0.99 (0.97)		**3.39 (<0.01)**
***How often have to adjust pace because of health***						
*A little/none of the time*			1.00		1.00	1.00
*Some of the time*			**1.35 (0.03)**		**1.54 (0.03)**	**1.56 (0.02)**
*All/most of the time*			0.75 (0.11)		**2.10 (0.01)**	**0.49 (0.01)**
***Do things to reduce stress***						
*Yes/sometimes*		1.00				1.00
*No*		**1.83 (0.01)**				**1.49 (0.04)**
***Try and stay connected to people***						
*Yes/sometimes*						1.00
*No*						**2.02 (0.01)**
***Ever used trial and error***						
*No/ don’t know/refused*	1.00	1.00			1.00	
*Yes/sometimes*	**1.71 (<0.01)**	**2.23 (0.05)**			**0.67 (0.03)**	
***How often do you feel angry about your health***						
*A little/none of the time*		1.00	1.00	1.00		1.00
*Some of the time*		**2.23 (<0.01)**	**1.28 (0.09)**	**1.55 (0.02)**		**2.41 (<0.01)**
*All/most of the time*		1.26 (0.53)	**2.12 (<0.01)**	**2.37 (0.01)**		**7.12 (<0.01)**
***Do you care about your health***						
*A little/none of the time*	1.00			1.00		
*Some of the time*	1.43 (0.21)			**2.09 (0.02)**		
*All/most of the time*	**1.89 (0.01)**			1.11 (0.69)		

## Discussion

The results of these analyses highlight firstly, the relationship between actual behaviour and perception of behaviour with large proportions of the population having an incorrect perception of their risk. Secondly, the analysis highlighted the proportions of people who worry about their health as a result of not undertaking the correct behaviour with substantial proportions of this population reporting high levels of worry. The results of the multivariable analyses highlight the similarities and dissimilarities between a wide range of demographic, socioeconomic and other related variables for the six key behavioural indicators. The multivariate analysis concentrated on those whose perception is that they undertake unhealthy behaviours, based on the premise that this perception is required before any behaviour change can be undertaken. If a stage of change model was employed these people would be in the contemplation and preparation stages [1]. What this analysis has shown is that there are clear demographic and health-related variables that are different between the groups who are, and are not, worried about the health effects of their actions.

One of the most striking features of the multivariate analyses was the markedly different profiles for different risk factors. Noticeable in these results is the ‘different’ profile for smokers (less likely to fit with the other risk factors) and the range of positive associations with BMI. This highlights the fact that campaigns need to be targeted differently depending upon the profile of the population who are most likely to act upon the message. As argued by others [26,27], the tailoring of specific messages to specific groups is an important endeavour to counteract the broad, population-wide, non-specific messages commonly used. There is a need to look past the demographic areas of research so that additional detail on the broader life and health context details are provided.

The most striking commonalities across the behaviours was age with all risk factors associated with at least one age group. The 45 to 54 year olds were most likely to have increased odds for each risk factor. This highlights the middle age groups as key targets for interventions, with those who are in the risk categories and are worried about the effect the risk factor is having on their health, being perfect targets for interventions. Other studies have found that midlife is an important time of life to make positive behavioural changes [28-30]. Interestingly, a trend was apparent for smoking and psychological distress with each younger age group more likely to have higher odds indicating that the young smokers and the younger persons with high levels of psychological distress are prime targets for interventions.

While research has highlighted the socio-economic differences apparent in risk behaviours with lower income groups more likely to be smokers [31], undertake less exercise [32], and have higher rates of obesity [33], this analysis showed that the relationship is not necessarily as straight forward as it seems. While our only measure of socio-economic status was annual household income, it was the middle household income level ($40,000 to $80,000 per year) who were more likely to be in the final models for BMI and high psychological distress indicting that campaigns targeting middle income levels for this risk factor should be considered. The lower income level (<$40,000) was also statistically significantly more likely to be included in the BMI model indicting that for BMI both lower income groups are also targets for intervention. In contrast, the middle income level was statistically significantly lower for physical inactivity indicting that this income group were less likely to be worried about their inactivity. No such clear message was apparent in our analysis for fruit and vegetable, alcohol and smoking with household income not included in the final models. Again the need for more detailed, topic-specific interventions are warranted.

A visit to a doctor was a variable included in the final model for BMI highlighting the important opportunity the general practitioner has in influencing these adults. Not surprisingly, smokers were significantly less likely to visit a doctor 10 or more times in the past year. This pattern was repeated for visits to other health professionals with smokers statistically significantly less likely to visit other health professionals while those at risk for low fruit and vegetable consumption and alcohol were statistically significantly more likely to visit other health professionals at least one to four times per year. Previous research has highlighted the important role that general practitioners and other medical specialists have in encouraging and influencing positive behavioural change of their patients [34,35], although concerns have been expressed on how successful the uptake of guidelines in this area have been [36].

Interestingly the overall health status variable was included in only three of the models (physical inactivity, smoking and high psychological distress) with higher odds for those respondents reporting fair/poor health. The variable that assessed anger with current health status was also included in these three models in addition to the fruit and vegetable model. While it is acknowledged that anger is associated with many chronic diseases including heart disease [37], depression and other mental health problems [38], diabetes [39], and arthritis [40] the relationship with risk factors has not been explored and highlights an area for further research.

One of the major strengths of this study is the use of a large randomly selected sample of the Australian population. The large sample size allows for greater generalisation of results. The weaknesses of this study include the cross-sectional nature of the data collection with the consequent inability to determine direction of effect. The reliance on self-report for some of the assessed variables is vulnerable to social desirability or other biased responses and is also a weakness of this study. In addition, sampling by telephone directory is likely to under sample some groups in the community. The study only involves community living adults and as such people living in supported accommodation such as aged care facilities would be missed from the sample. The response rate of nearly 44% is acceptable for this type of survey but the potential for survey non-response bias is acknowledged. Response rates are declining in surveys based on all forms of interviewing [41,42] as people have become more active in protecting their privacy. The growth of telemarketing has disillusioned the community and diminished the success of legitimate social science research by means of telephone-based surveys. In addition, the increased use of mobile telephones and decreased use of land-lines could result in an under-representation of younger respondents (with younger persons more likely to have mobile telephones only and hence be excluded from sampling frames based on listed telephone numbers). Up to 5% of telephone calls made were on mobile telephones (those that are listed in the EWP or those that are obtained when contact is made with the household).

Other weaknesses of the study are the lack of validation of some of the variables and the fact that these data elements were collected with a range of other variables that were not included in the analysis. This exclusion of these other variables did not allow for consideration of potential confounders. Only using questions pertaining to fruit and vegetable consumption to represent a balanced diet could also be seen as a weakness of the study. Notwithstanding these weaknesses, the overall prevalence estimates obtained from this survey are in line with state and national estimates indicating a non-biased sample.

## Conclusion

Research is needed on the relationship between worry, perceived risk and actual behaviours rather than behavioural intentions [8] and this study has assisted in this development. Further research could develop this relationship between perceived risk, worry and actual behavioural by assessing intentions to change.

While there is a known cluster of risk factors [43] the characteristics associated with those who worry about the risk factor, as shown in this study, vary remarkably. As argued by Baron et al. [5] some people over-worry or worry about the wrong aspects and this study has shown that much of the population are worrying about the health affect of a behaviour they are actually undertaking adequately. This research has determined a unique way of providing evidence for health promotion campaigns centred on reducing inappropriate health behaviours.

## Abbreviations

BMI: Body Mass Index; CATI: Computer Assisted Telephone Interviewing; EWP: Electronic White Pages; K10: Kessler 10; OR: Odds ratio; SPSS: Statistical Package for Social Sciences.

## Competing interests

The authors declare that they have no competing interests.

## Authors’ contribution

AWT and KP were responsible for the conception and design of the study. SF conducted the analysis. AWT drafted the manuscript. All authors had full access to the reports and tables and provide advice on analysis and interpretation of data. All authors critically reviewed the draft versions and approved the final version of the manuscript.

## Pre-publication history

The pre-publication history for this paper can be accessed here:

http://www.biomedcentral.com/1471-2458/13/120/prepub
